# Land Use and Land Cover Stability Across Protection Regimes in a Tropical River Basin

**DOI:** 10.1007/s00267-026-02459-4

**Published:** 2026-04-21

**Authors:** Marcelo Henrique Schmitz, Levi Carina Terribile, Vitor das Neves Cardoso, Roniel Freitas-Oliveira, Patrick Thomaz de Aquino Martins

**Affiliations:** 1 Laboratory of Geomatics and Marine Systems (GEOMar), Marine Management, Ecology, and Technology Group (GTMar), State University of Santa Catarina, Laguna, Santa Catarina Brazil; 2 Macroecology Laboratory, Federal University of Jataí, Jataí, Brazil; 3 Phytoplankton and Marine Microorganisms Laboratory, Institute of Oceanography, Federal University of Rio Grande—FURG, Rio Grande, RS Brazil; 4 Laboratório de Geoprocessamento, Campus Nordeste, State University of Goiás, Formosa, GO Brazil

**Keywords:** Anthropogenic Pressures, Conservation Effectiveness, Deforestation, Geospatial analysis, MATOPIBA, Sustainability

## Abstract

Tropical frontiers are undergoing rapid land transformations that threaten biodiversity and ecosystem resilience. Using annual land use and land cover (LULC) data from 1985 to 2022 derived from MapBiomas Collection 8, we assessed cumulative landscape stability across three legally defined protection levels in the Araguaia River Basin, a major South American agricultural frontier. Over the study period, unprotected areas experienced the largest net losses of Natural Forest and Savanna and the lowest landscape stability, with less than 50% of the area remaining unchanged. In contrast, fully protected areas showed the highest stability, with more than 80% of their area presenting zero accumulated transitions. Sustainable use areas displayed intermediate dynamics. Environmental degradation transitions were consistently more frequent in unprotected zones, whereas restoration transitions were spatially heterogeneous and co-occurred with degradation hotspots. These results indicate that landscape dynamics differ markedly across protection categories, reflecting distinct long-term land-use spatial patterns. By providing a spatially explicit cumulative assessment over nearly four decades, this study offers descriptive evidence relevant to conservation planning and land management in rapidly transforming tropical frontiers.

## Introduction

Global trends in land use and land cover (LULC) change, particularly deforestation, agricultural expansion, and urbanization, have profoundly altered biodiversity, ecosystem services, and climate regulation (Vitousek et al. 1997; Leberger et al. 2020). Beyond documenting net area losses, annual remote-sensing time series allow the identification of long-term land-cover trajectories, revealing where landscapes remain stable and where repeated transformations occur. Such spatially explicit assessments are especially relevant in rapidly expanding frontiers, where legally defined protection categories coexist with intense anthropogenic pressures. In this context, quantifying land-cover transitions provides a consistent basis for comparing landscape stability across protection levels and for evaluating how land-use dynamics vary under different regulatory frameworks (Martins et al. 2021; Schmitz et al. 2023).

One of the principal responses to environmental degradation has been the establishment of legally protected areas (PAs) (Azevedo-Santos et al. 2017; Guerra et al. 2019; Leberger et al. 2020). Beyond restricting specific land uses, protected areas function as regulatory instruments that structure territorial planning and conservation management (IUCN 1994; Gill et al. 2024). Different protection categories impose varying degrees of regulation, which may influence landscape in distinct ways. Fully protected areas prioritize biodiversity conservation by limiting extractive activities and maintaining habitat continuity (IUCN 1994; Brazil 2000; Leberger et al. 2020), whereas sustainable use areas seek to reconcile conservation objectives with regulated resource use and long-term landscape management (IUCN 1994; Brazil 2000; Ruggiero et al. 2022).

Measuring the effectiveness of protected areas remains complex because LULC dynamics are shaped by both natural processes, such as ecological succession and hydrological cycles, and anthropogenic pressures, including agricultural expansion and illegal logging (Guerra et al. 2019; Metzger et al. 2019; Conceição et al. 2022). In this context, remote-sensing time series provide spatially explicit baselines and long-term indicators of land-cover persistence. One valuable metric in this context is landscape stability, which refers to the consistency of land cover types over time (López et al. 2013; Guerra et al. 2019; Schmitz et al. 2023). In this study, stability is defined as the persistence of a land-cover category at the pixel level throughout the entire time series. High landscape stability within PAs denotes limited land-cover dynamics, which may result from effective conservation management or from the persistence of previously modified landscapes. Therefore, stability should be interpreted in conjunction with land-cover composition, historical trajectories, and contextual pressures to accurately assess ecological integrity (Schmitz et al. 2023).

In Brazil, protected areas serve as key conservation instruments amid ongoing environmental pressures, particularly deforestation (Brazil 2000; MapBiomas 2024). They encompass diverse ecosystems, including the Amazon rainforest, Atlantic Forest, and Cerrado savannas, which are central to biodiversity conservation and ecosystem service provision (Metzger et al. 2019; de Melo and Martins 2020; Conceição et al. 2022). However, limited biodiversity monitoring in many regions constrains direct assessments of conservation outcomes (Azevedo-Santos et al. 2017; Alves et al. 2019; Correa et al. 2020). In such contexts, LULC stability derived from remote sensing is frequently used as a spatial proxy to examine long-term land-cover persistence, as well as protected-area effectiveness, particularly in regions where persistent pressures such as illegal logging, agricultural expansion, and institutional constraints continue to influence landscape dynamics (Latrubesse et al. 2019; Pelicice and Castello 2021; Ruggiero et al. 2022).

Protected areas are not randomly distributed across landscapes and often reflect pre-existing gradients of accessibility, governance structures, and human pressure (Joppa and Pfaff 2009; Pfaff et al. 2014; Herrera et al. 2019; Keles et al. 2020, 2023). Recognizing this complexity, we focus on a deforestation frontier where legal protection categories operate within broadly comparable regional pressures, enabling a spatially explicit assessment of landscape stability under different levels of regulation.

The Araguaia River basin, located within Brazil’s agricultural frontier, provides an appropriate case study for examining these dynamics. Covering approximately 380,000 km², and encompassing savannas, forests, and extensive floodplains, the basin has experienced rapid agricultural expansion over recent decades (Martins et al. 2021; Pelicice et al. 2021). Although stricter land-use regulation is generally associated with lower anthropogenic pressure (Guerra et al. 2019; Jesus et al. 2020; Keles et al. 2023; Schmitz et al. 2023), ecotonal and floodplain systems may also exhibit elevated natural dynamics. In the Araguaia basin, seasonal flooding and ecological succession contribute to land-cover transitions among natural categories (Homeier et al. 2017; Petsch et al. 2023), which must be considered when interpreting stability patterns.

Building on these considerations, we addressed the following questions: (1) how general LULC area changes vary across different levels of legal protection in the Araguaia River basin; (2) whether areas under stricter legal protection exhibit higher stability than areas with less land-use regulation; and (3) whether environmental degradation and restoration transitions differ across protection levels. Based on previous empirical studies, we predicted that fully protected areas would exhibit higher stability compared to sustainable use and non-protected areas.

## Methods

### Study Area

**Fig. 1:**
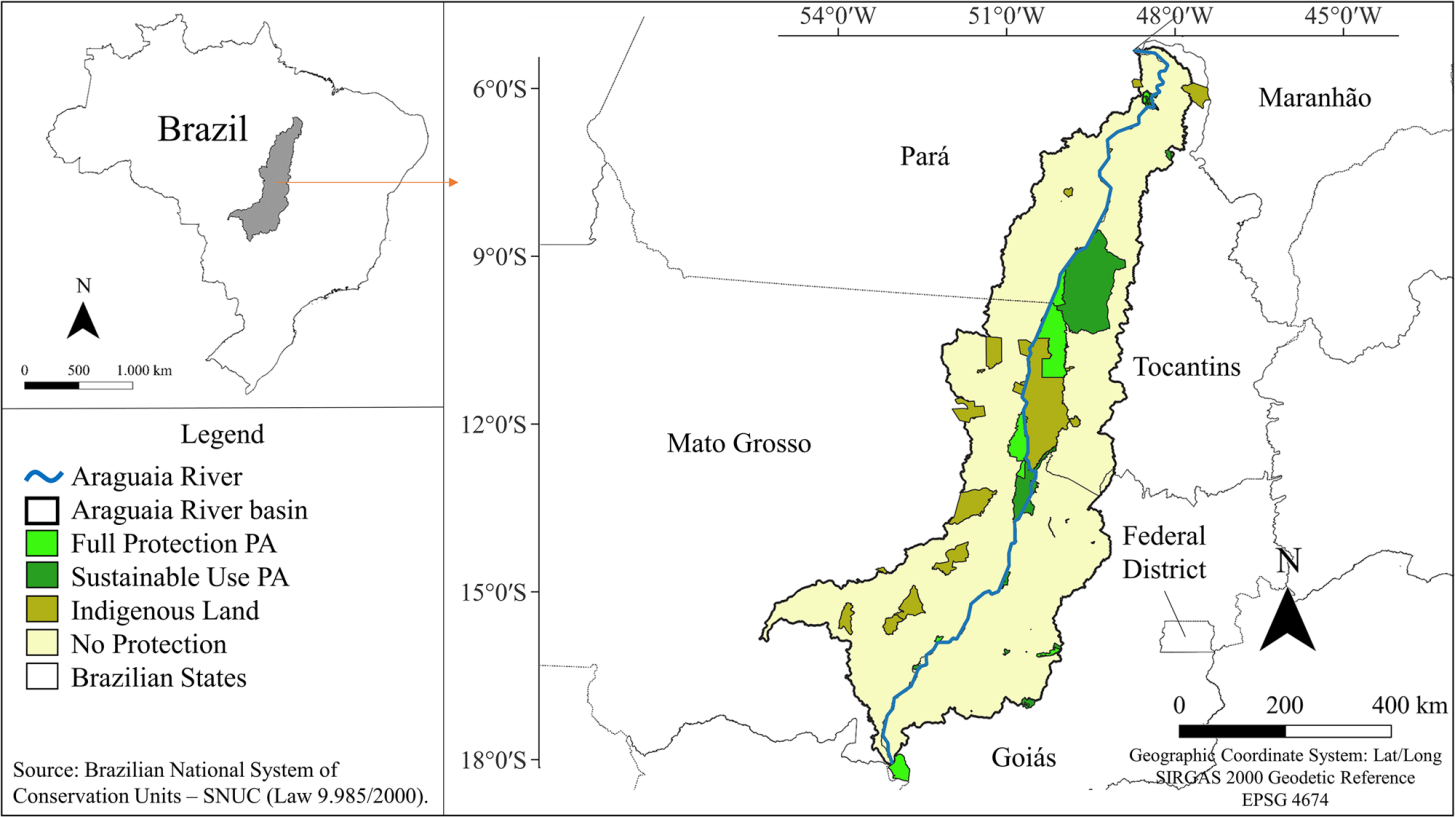
Map of the Araguaia River basin highlighting the three protection levels: Full Protection, Sustainable Use (Comprised by Sustainable Use PAs and Indigenous Lands), and No Protection

The Araguaia River basin covers approximately 380,000 km² in central Brazil and spans a major Amazon–Cerrado ecotone characterized by pronounced climatic, geomorphological, and socio-economic gradients (Martins et al. 2021; Pelicice et al. 2021). Extending across Goiás, Mato Grosso, Tocantins, and Pará, the basin integrates extensive floodplains, savannas, and forest formations (Fig. 1). Over recent decades, it has become embedded within Brazil’s agricultural frontier, experiencing rapid expansion of cattle ranching and mechanized soybean production, particularly in its southern and central sectors (Polizel et al. 2021; MapBiomas 2024). The basin’s large floodplain system introduces strong seasonal hydrological dynamics and natural vegetation shifts that interact with anthropogenic pressures, contributing to heterogeneous LULC trajectories across the region (Homeier et al. 2017; Petsch et al. 2023).

### Data

We obtained annual LULC raster data for the years 1985 to 2022 from MapBiomas Collection 8, which produces annual land-use and land-cover maps using supervised machine-learning classification primarily applied to Landsat imagery within the Google Earth Engine environment (Souza et al. 2020; MapBiomas, 2023). All rasters were provided in SIRGAS 2000 (EPSG 4674). Spatial boundaries of protected areas and Indigenous Lands were obtained from the Brazilian National Registry of Conservation Units (Ministry of Environment and Climate Change 2023).

### LULC Processing and Reclassification

Original MapBiomas categories were reclassified into 10 representative classes: Natural Forest, Savanna, Wetland, Grassland, Pasture, Agriculture, Urban Area, Non-Vegetated Area, Other Human Activity, and Water. The full crosswalk between original MapBiomas classes and the adopted categories is provided in Table S1 (Supplementary Material). From the reclassified dataset, overall LULC area changes were extracted at the basin level (objective 1).

For each pixel, we calculated the number of categorical transitions between consecutive years from 1985 to 2022, hereafter referred to as accumulated changes. A transition was defined as any shift from one class to another between successive annual maps.

A second reclassification grouped the 10 classes into two broader categories: Natural and Anthropogenic. Based on this grouping, we quantified, per pixel, the number of transitions representing environmental degradation and environmental restoration. Environmental degradation was operationally defined as the conversion of a natural land-cover class to an anthropogenic class between consecutive years, reflecting structural land-cover transformation rather than ecological condition. Environmental restoration was defined as the reversion from an anthropogenic to a natural class in the time series.

### Comparison Across Protection Levels

Three protection levels were considered: Full Protection, Sustainable Use, and No Protection, following the Brazilian National System of Conservation Units (SNUC; Law 9.985/2000). All protected areas overlapping the basin were grouped according to their legal designation. The year of establishment of each protected area and Indigenous Land was compiled to provide temporal context for the analysis. Because these areas were created at different times, these dates are reported in Tables S2, S3, and S4 (Supplementary Material) and should be considered when interpreting cumulative landscape dynamics over the 1985–2022 period. Full Protection included 11 units (7 State Parks, 2 National Parks, 1 Municipal Park, and 1 State Wildlife Refuge). Sustainable Use included 20 sustainable use units and 28 Indigenous Lands, grouped due to their legally recognized territorial status and regulated resource-use framework. Units within each category were merged into a single polygon. In cases of spatial overlap, Full Protection units were prioritized over Sustainable Use to avoid double counting. The No Protection category comprised the remaining basin area.

Landscape stability (objective 2) was quantified using the total number of accumulated changes per pixel over the study period. For each protection level, we summarized the proportion of total area exhibiting 0, 1, 2, 3, 4–6, 7–10, or more than 11 changes. Short-term oscillations were not filtered and are considered part of the cumulative signal.

To visualize the spatial distribution of restoration and degradation transitions, we applied a quartic kernel density analysis with a 1000 m search radius. This bandwidth represents an intermediate scale relative to the 30 m spatial resolution, approximately 33 pixels, allowing detection of local clustering without excessive smoothing. The kernel output was used descriptively for spatial visualization only.

Differences in restoration and degradation among protection levels (objective 3) were assessed using a resampling-based non-parametric approach. In each of 1000 iterations, 1000 pixels were randomly sampled from each protection level to ensure balanced comparison while maintaining computational feasibility. For each iteration, a Kruskal–Wallis test was performed, followed by pairwise Mann–Whitney U tests. The distribution of H statistics across iterations was used to evaluate the robustness of differences among protection levels rather than relying on single *p* values. A Shapiro–Wilk test was applied to assess whether the mean adequately represented the distribution of H statistics. Because spatial dependence is not fully eliminated, results are interpreted as comparative and descriptive rather than causal.

All spatial analyses were conducted in QGIS 3.2. Statistical analyses and plots were produced in R 4.1.

## Results

**Fig. 2:**
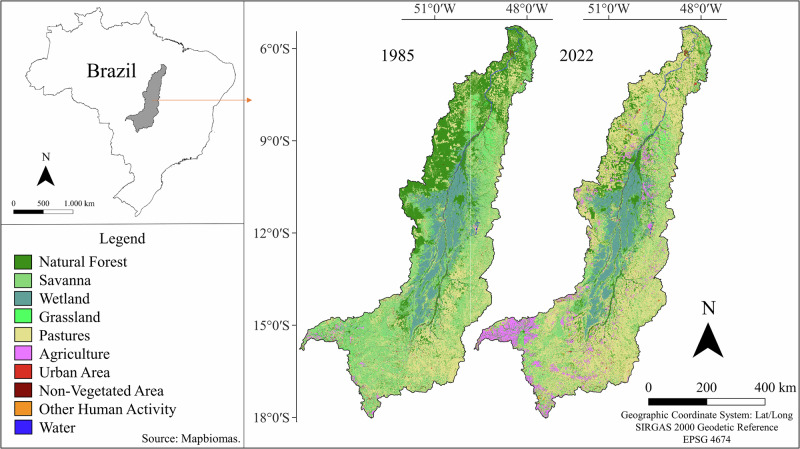
Comparison of land use and land cover in the Araguaia River basin between the years 1985 and 2022

**Fig. 3:**
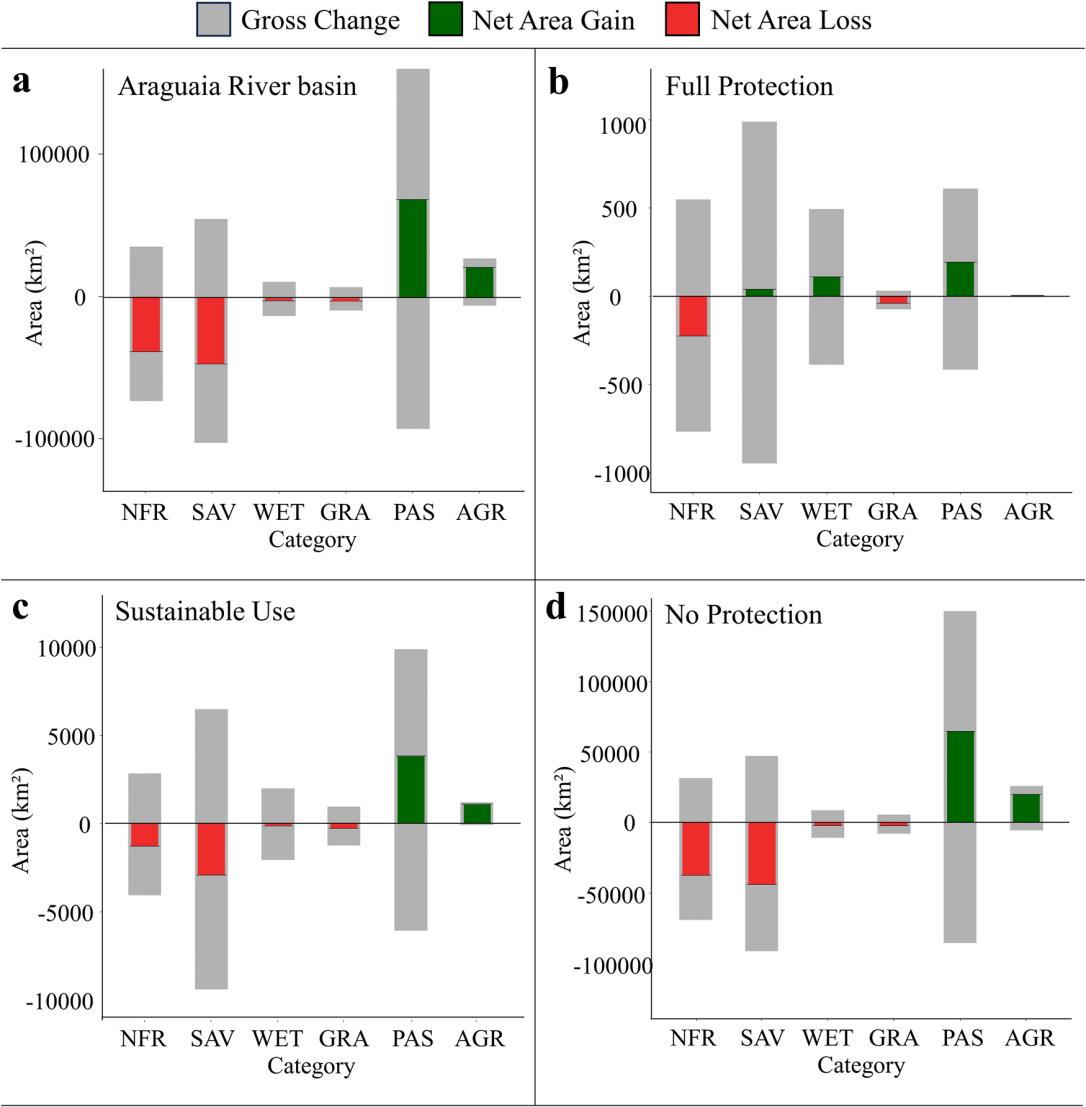
Diagram showing the gross change (gray) and the net gains (green) and losses (red) of area (km²) for the main land use and cover categories in the Araguaia River basin (**a**), Full protection level (**b**), Sustainable Use level (**c**), and No Protection level (**d**) from 1985 to 2022. Categories: NFR Natural Forest, SAV Savanna, WET Wetland, GRA Grassland, PAS Pasture, AGR Agriculture. The categories Urban Area, Non-Vegetated Area, Other Human Activity, and Water presented low area values and were omitted

The comparison of land use and land cover in the Araguaia River basin between 1985 and 2022 revealed significant transformations, particularly the reduction of natural forests and savannas, alongside the expansion of agricultural activities and pastures (Fig. 2). In 1985, natural forests predominantly covered the basin, especially in the northern region. However, by 2022, these forests had substantially diminished, giving way to increased areas of pastures, mainly in the northern region, and agricultural lands, particularly in the southern and central parts of the basin. Quantitatively, this trend was marked by significant net losses in Natural Forest and Savanna areas and substantial net gains in Pasture, as observed at the basin level (Fig. 3a).

This pattern was consistent across different protection levels, although with varying magnitudes. In the Full Protection level (Fig. 3b), there were decreases in Natural Forest and Grasslands, while Wetlands and Savannas showed small net area gains. In the Sustainable Use level (Fig. 3c), moderate net losses in Natural Forest and Savanna were noted, though less pronounced than in areas under No Protection (Fig. 3d). The No Protection areas exhibited the most substantial transformations, with extensive gross changes, large net losses in Natural Forest and Savanna, and significant net gains in Pasture and Agriculture. Categories such as Urban Area, Non-Vegetated Area, Other Human Activity, and Water were omitted due to their minimal area changes.

**Fig. 4:**
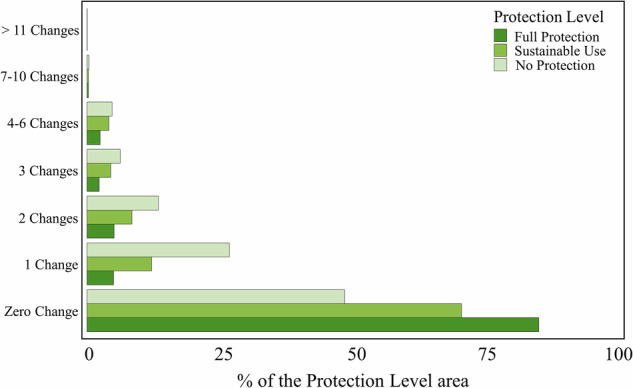
Bar plots of the accumulated changes during the period of 1985–2022 indicating the percentage of the total area in the three Protection Levels: Full Protection, Sustainable Use, and No Protection

Full Protection exhibited the highest stability, with over 80% of the area showing zero changes (Fig. 4). In contrast, areas with No Protection had the lowest stability, with less than 50% of the area experiencing zero changes, hence a significant proportion of the landscape underwent one or more changes.

Areas under the Sustainable Use level showed intermediate levels of stability, with a higher percentage of the area experiencing zero changes compared to No Protection but lower than Full Protection. The frequency of one to three changes is higher in Sustainable Use areas compared to Full Protection areas, indicating moderate landscape stability. The data also highlight that areas with No Protection experienced the highest frequency of multiple changes (four or more), reflecting more intense land use dynamics. These results show that Full Protection areas had the highest landscape stability, whereas Sustainable Use areas displayed intermediate levels of change relative to the other categories.

**Fig. 5:**
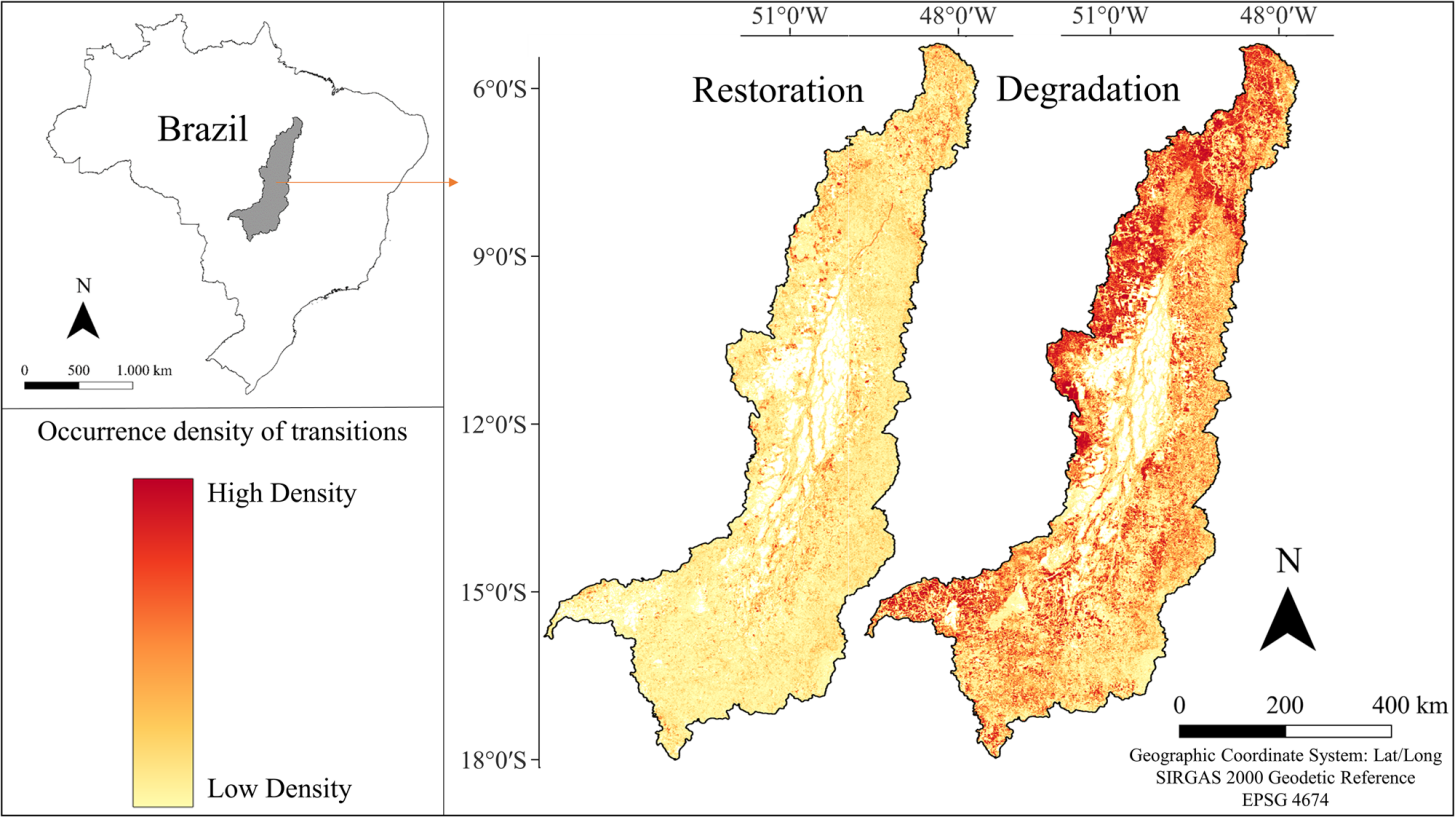
Heatmaps (Kernel density) indicating areas with high (red) and low (beige) density of occurrence of pixels containing environmental restoration (left) and degradation (right) transitions in the Araguaia River basin between 1985 and 2022

The spatial distribution of the density of restoration transitions in the Araguaia River basin indicates overall low-density values (Fig. 5). Conversely, the map highlighting areas of environmental degradation was marked by high-density areas, particularly concentrated in the central and northern regions of the basin, consistent with areas of low anthropogenic stability. The contrast between the two maps underscores the spatial heterogeneity of land cover dynamics within the basin.

**Fig. 6:**
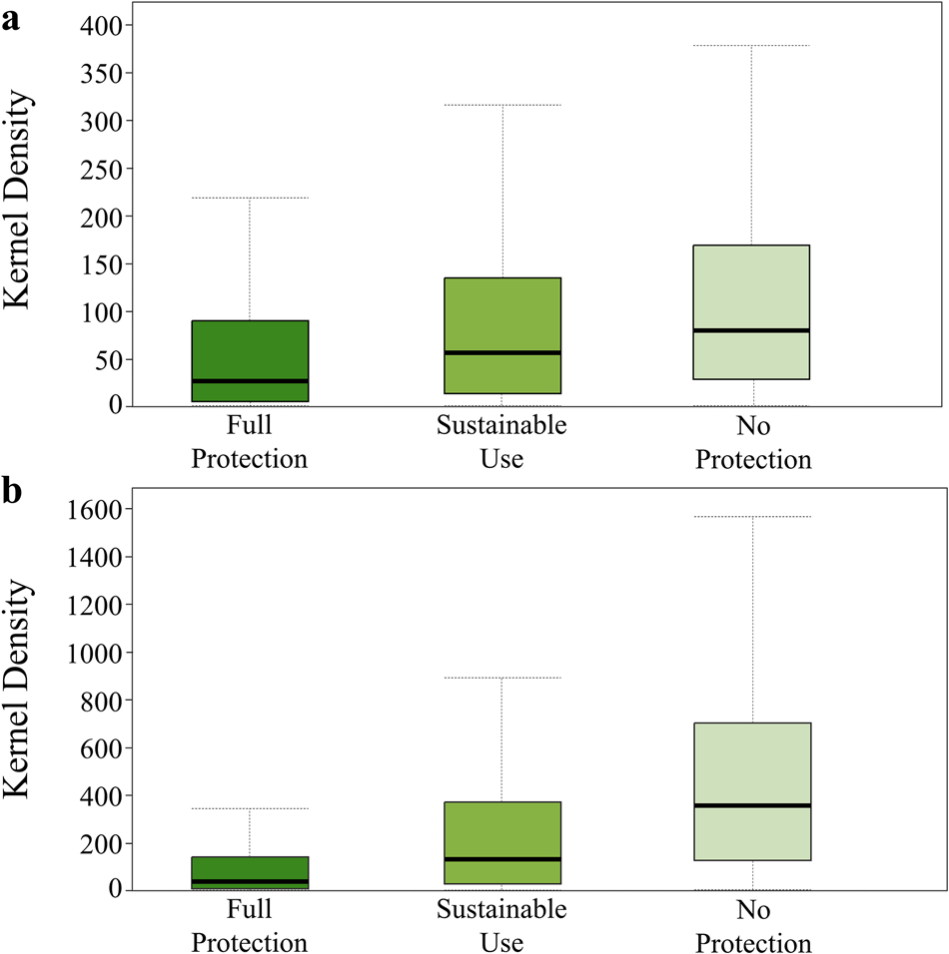
Boxplots showing the data patterns of accumulated environmental restoration (**a**) and degradation (**b**) across protection categories

The analysis of the kernel density data also shows clear differences in both environmental restoration and degradation across protection categories (Fig. 6). For environmental restoration, areas under Full Protection exhibited the lowest values and variability, while No Protection areas showed the highest restoration values and dispersion. In contrast, environmental degradation was markedly higher in No Protection areas, with Full Protection zones presenting the lowest degradation levels. These patterns show that degradation and restoration dynamics differ across protection categories, with lower degradation values observed in Full Protection areas and higher and more variable values in No Protection areas.

The resampling-based analysis revealed consistent differences among protection levels for both restoration and degradation (Fig. S5, Supplementary Material). Across 1000 iterations, all Kruskal–Wallis tests were statistically significant (*p* < 0.05 in 100% of cases), indicating systematic separation among categories. For degradation, the distribution of H statistics did not deviate from normality (Shapiro–Wilk *p* = 0.3344), and the mean H value was 304.95, indicating strong divergence among protection levels. For restoration, H values deviated from normality (Shapiro–Wilk *p* < 0.001); therefore, both mean (42.93) and median (41.93) are reported, indicating moderate but consistent separation. The consistent significance across iterations demonstrates the robustness of these differences under balanced resampling. Given residual spatial dependence, results are interpreted as comparative and descriptive rather than causal.

## Discussion

Our results for land use and land cover changes in the Araguaia River basin between 1985 and 2022 revealed substantial transformations, particularly the reduction of natural forests and the expansion of agricultural activities. A decrease in natural categories was observed across all protection levels, with the most substantial changes occurring in areas without protection, which also showed the highest levels of environmental degradation and restoration transitions. In contrast, fully protected areas exhibited the greatest stability, with minimal landscape changes, while sustainable use areas presented intermediate patterns. The spatial distribution of restoration and degradation densities highlights the concentration of environmental change in unprotected areas. Overall, these findings indicate that land-cover dynamics and landscape stability differ consistently across protection levels.

Comparing LULC changes across protection levels provides a comparative perspective on land-cover dynamics within the Amazon–Cerrado transition zone, characterized by forests, savannas, and extensive floodplains. Consistent with other ecotonal regions (Metzger et al. 2019; Jesus et al. 2020; Keles et al. 2023; Schmitz et al. 2023), fully protected areas showed lower transition frequencies and smaller net losses of natural vegetation. In contrast, unprotected areas concentrated the most substantial transformations, particularly forest and savanna conversion to pasture and agriculture, especially in sectors more exposed to agricultural expansion and infrastructure development.

Sustainable use areas showed intermediate responses, suggesting intermediate land-cover dynamics under regulated-use categories in the context of strong regional economic pressures. Even fully protected areas exhibited declines in natural forests and grasslands, along with marked savanna transitions. The magnitude of these changes appears higher than in more consolidated forest regions, suggesting that ecotonal and savanna-dominated systems occupying much of the basin may be more susceptible to land-use pressure. Together, these patterns indicate that transition intensity differs across protection categories, while broader frontier dynamics and biome-specific structural conditions remain central to understanding landscape change in the region (Correa et al. 2020; Caballero et al. 2023; MapBiomas, 2024).

Deforestation in the Amazon and Cerrado biomes remains a major environmental issue largely driven by agribusiness expansion (da Cruz et al., 2021; Caballero et al. 2023; Haddad et al., 2024; MapBiomas, 2024). Large-scale agriculture and cattle ranching have driven land conversion in central Brazil in recent decades. The forest and savanna losses observed in non-protected areas align with documented pasture expansion and soybean-driven frontier dynamics (Latrubesse et al. 2019; Diniz-Filho et al. 2020; da Cruz et al., 2021; Polizel et al. 2021).

The concentration of transitions toward pasture and agriculture reflects these regional production systems. Although protected areas are often associated with lower deforestation rates (Alves et al. 2019; de Melo and Martins 2020; Ruggiero et al. 2022), continued transitions within protected categories suggest that enforcement constraints and illegal activities still shape land-use trajectories.

Assessing protected-area performance in the basin requires recognizing that LULC transitions may reflect both anthropogenic pressures and natural ecological dynamics. In the Araguaia floodplain, seasonal flooding and ecological succession drive turnover among vegetation types (Homeier et al. 2017; Petsch et al. 2023), which are recorded as transitions among natural classes. Lower stability therefore does not necessarily indicate ecological degradation.

Even so, fully protected units, many within the floodplain, showed higher stability than non-protected areas despite strong fluvial dynamism. This pattern is consistent with lower levels of structural land-cover conversion observed in fully protected areas despite ecological variability, consistent with broader regional assessments (Metzger et al. 2019; Conceição et al. 2022). The stability metric does not distinguish natural from anthropogenic drivers within natural categories, and no explicit driver attribution was conducted. Results should therefore be interpreted as patterns of land-cover persistence and conversion rather than causal effects. Future analyses incorporating hydrological or other spatial covariates could refine this distinction.

Fully protected areas in the basin showed the highest stability, reflected in a greater proportion of zero-change areas, a pattern also reported in other Brazilian basins (Schmitz et al. 2023). These areas are governed by stricter regulations aimed at preserving natural ecosystems and biodiversity. Sustainable use areas displayed intermediate stability, likely reflecting the Brazilian protected areas framework, which permits regulated human activities, including sustainable use units and Indigenous Lands (Brazil 2000; de Melo and Martins, 2020; Fa et al. 2020; Ruggiero et al. 2022). Indigenous Lands have been shown to support biodiversity conservation and climate change mitigation by reducing deforestation within their territories (Soares-Filho et al. 2010; Correa et al. 2020; Siqueira-Gay et al. 2020).

Areas without protection showed higher landscape dynamics and frequent LULC transitions, particularly those linked to agricultural activities (Martins et al. 2021; Pelicice et al. 2021; Haddad et al., 2024; MapBiomas 2024). These regions had the highest density of degradation and restoration transitions. Their spatial co-occurrence suggests recurrent land-use turnover, potentially associated with short-term management cycles, pasture–crop rotation, or abandonment–recovery processes.

Although the available LULC data do not allow direct attribution of causal mechanisms, this pattern aligns with disturbance and recovery dynamics described in agricultural frontier landscapes. Transitions from natural vegetation to pasture and agriculture were concentrated in sectors influenced by Brazil’s agricultural frontier, including areas associated with MATOPIBA (Pelicice et al. 2021; Polizel et al. 2021; Agostinho et al. 2023). The net losses of forests and savannas and high gross-change values in non-protected areas are consistent with documented pasture expansion and soybean-driven land-use restructuring in central Brazil (Latrubesse et al. 2019; Diniz-Filho et al. 2020; Polizel et al. 2021).

While this analysis does not allow direct attribution of transitions to specific policy instruments, the overlap between high transition densities and frontier zones suggests that agricultural intensification operates alongside the land-cover dynamics observed in the basin. The co-occurrence of degradation and restoration transitions may reflect cycles of land conversion, temporary abandonment, and reoccupation, as described in rapidly transforming agricultural landscapes (Pelicice et al. 2021; Agostinho et al. 2023).

This study relies on spatial comparisons among protection categories within a single frontier context and does not implement matching or before–after–control designs. Protected areas were established at different times, and consistent pre-designation baselines are not available across categories. Consequently, the observed differences should be interpreted as spatial associations within the cumulative 1985 to 2022 trajectory rather than as causal effects of legal protection. Even so, the consistency and magnitude of these spatial patterns provide robust evidence of differentiated landscape trajectories across protection regimes.

Protected areas in Brazil are established under complex social, political, and ecological criteria that may introduce location bias (Joppa and Pfaff 2009; Pfaff et al. 2014). Stricter categories often coincide with areas of lower historical anthropogenic pressure, partially explaining their higher stability (Herrera et al. 2019; Correa et al. 2020; Keles et al. 2023). Accordingly, the results should be interpreted as descriptive evidence of land-use dynamics across distinct legal protection categories rather than as a direct assessment of conservation effectiveness.

The observed patterns highlight the persistence of natural forests and savannas within stricter protection categories. The results show lower land-use conversion rates in these areas, while sustainable use and unprotected lands exhibit greater variability and pressure. Similar patterns have been documented in tropical forest frontiers, where stricter categories are associated with reduced deforestation, although outcomes depend on institutional and spatial context (Azevedo-Santos et al. 2017; Ruggiero et al. 2022). Maintaining regulatory consistency and monitoring capacity remains relevant in landscapes undergoing rapid agricultural expansion (Agrawal et al. 2022; Caballero et al. 2023).

## Conclusion

This study provides a long-term, spatially explicit evaluation of land-use and land-cover dynamics across legally defined protection categories in the Araguaia River basin, a region undergoing sustained agricultural frontier expansion. By systematically comparing fully protected areas, sustainable use areas, and unprotected lands over nearly four decades, we demonstrate that landscape stability is unevenly distributed and consistently associated with protection categories. Fully protected areas exhibited persistent structural stability, whereas unprotected areas showed higher rates of conversion and cumulative transformation. Sustainable use areas displayed intermediate dynamics, indicating variable land-use dynamics across categories.

Rather than offering a general statement about protected areas, this analysis quantifies how differential stability patterns emerge within a frontier context where conservation and production systems increasingly intersect. The integration of consistent multi-decadal land-cover mapping with categorical comparison provides an empirical baseline for assessing long-term spatial trajectories in tropical basins subject to rapid change. By explicitly documenting where conversion pressures concentrate and where stability persists, the study advances understanding of how landscape-scale land-use outcomes differ across legal protection categories under continued development pressure.

## Supplementary information

Supplementary Material

## Data Availability

Data will be made available on reasonable request.
