# Supplementary material for: Land Use and Land Cover Stability Across Protection Regimes in a Tropical River Basin
Source: Environ Manage. 2026 Apr 21;76(5):165. doi: 10.1007/s00267-026-02459-4 (PMC13099672; doi:10.1007/s00267-026-02459-4)
Supplement: Supplementary file 1 — Supplementary Material [file 267_2026_2459_MOESM1_ESM.pdf]

Land use and land cover stability across protection regimes in a tropical river basin

Supplementary Material.

Table S1. Reclassification rules for land use and land cover categories.

Mapbiomas Category	Mapbiomas Description	New Category	New Description
3	Forest Formation	1	Natural Forest
4	Savanna Formation	2	Savanna
6	Flooded Forest (beta)	1	Natural Forest
9	Forestry	9	Other Human Activity
11	Flooded Grassland and Wetland	3	Wetland
12	Grassland Formation	4	Grassland
15	Pasture	5	Pastures
20	Sugar Cane	6	Agriculture
21	Mosaic of Uses	5	Pastures
24	Urban Area	7	Urban Area
25	Other Non-Vegetated Areas	8	Non-Vegetated Area
29	Rock Outcrop	8	Non-Vegetated Area
30	Mining	9	Other Human Activity
33	River, Lake, and Ocean	10	Water
39	Soybean	6	Agriculture
40	Rice	6	Agriculture
41	Other Temporary Crops	6	Agriculture
46	Coffee	6	Agriculture
48	Other Permanent Crops	6	Agriculture
62	Cotton (beta)	6	Agriculture

Table S2. Special areas included in the "Full Protection" level. Items are organized by size.

Name	Instance	Creation	Area (km²)
Parque Nacional do Araguaia	National	1959	5,555.015
Parque Estadual do Araguaia	State	2001	2,299.201
Parque Nacional das Emas	National	1961	1,327.847
Parque Estadual do Cantão	State	1998	1,004.132
Refúgio de Vida Silvestre Corixão Da Mata Azul	State	2001	357.083
Parque Estadual da Serra Dourada	State	2003	286.435
Parque Estadual da Serra Dos Martírios/Andorinhas	State	1996	250.318
Parque Estadual Serra Azul	State	1994	110.066
Parque Estadual do Araguaia	State	2002	46.627
Parque Estadual de Paraúna	State	2002	33.351
Parque Natural Municipal de Redenção	Municipal	2020	0.164

Table S3. Special areas included in the "Sustainable Use" level. Items are organized by size.

Name	Instance	Creation	Area (km²)
Área de Proteção Ambiental Ilha do Bananal/Cantão	State	1997	15,700.104
Área de Proteção Ambiental Meandros do Araguaia	National	1998	3,591.896
Área de Proteção Ambiental da Serra Dourada	State	1998	370.400
Área de Proteção Ambiental da Serra das Galés e da Portaria	State	2002	319.869

Área de Proteção Ambiental de São Geraldo do Araguaia	State	1996	267.033
Floresta Estadual do Araguaia	State	2002	223.378
Área de Proteção Ambiental Lago de Santa Isabel	State	2002	185.856
Reserva Extrativista Lago do Cedro	National	2006	171.793
Área de Proteção Ambiental das Nascentes de Araguaína	State	1999	155.971
Área de Proteção Ambiental do Encantado	State	2003	94.445
Área de Proteção Ambiental Fazenda Nova	Municipal	2018	71.787
Reserva Particular do Patrimônio Natural Bico do Javaés	National	2011	27.620
Área de Proteção Ambiental do Fanha	Municipal	2018	15.810
Área de Proteção Ambiental Água Fria	Municipal	2021	13.316
Reserva Particular do Patrimônio Natural Sonhada	National	2010	8.870
Área de Proteção Ambiental Rio Palmeiral	Municipal	2015	6.144
Área de Proteção Ambiental de Caiaponia	Municipal	2021	3.413
Reserva Particular do Patrimônio Natural Nascentes do Rio Araguaia	National	2012	3.901
Área de Proteção Ambiental Novo Brasil	Municipal	2021	1.211
Área de Relevante Interesse Ecológico Águas de São João	State	2000	0.245
Área de Proteção Ambiental Ilha do Bananal/Cantão	State	1997	15700.104

Table S4. Indigenous lands included in the “Sustainable Use” level. Items are organized by size.

**Name**	**Instance**	**Officialization**	**Area (km²)**
Parque Indígena do Araguaia	Indigenous Land	1971	13789.226

Inawebohona	Indigenous Land	2006	3799.208
Pimentel Barbosa	Indigenous Land	1986	3305.623
Utaria Wyhyna/Iròdu Iràna	Indigenous Land	2010	1868.708
Areões	Indigenous Land	1996	1810.124
São Marcos - MT	Indigenous Land	1975	1730.319
Urubu Branco	Indigenous Land	1998	1679.714
Maraiwatsede	Indigenous Land	1998	1656.216
Apinayé	Indigenous Land	1997	1452.354
Sangradouro/Volta Grande	Indigenous Land	1991	1027.517
Merure	Indigenous Land	1987	826.251
Tapirapé/Karajá	Indigenous Land	1983	679.798
Cacique Fontoura	Indigenous Land	2024	332.105
Taego Ãwa	Indigenous Land	2016	285.249
Sororó	Indigenous Land	1983	261.348
Las Casas	Indigenous Land	2009	216.143
Chão Preto	Indigenous Land	2001	125.895
Krahó-Kanela	Indigenous Land	2006	77.209
São Domingos - MT	Indigenous Land	1991	61.666
Krenrehé	Indigenous Land	2014	54.221
Xambioá	Indigenous Land	1997	39.174
Carretão I	Indigenous Land	1990	16.873

Karajá Santana do Araguaia	Indigenous Land	1991	14.671
Karajá de Aruanã II	Indigenous Land	1998	9.583
Karajá de Aruanã III	Indigenous Land	2000	7.281
Maranduba	Indigenous Land	2005	3.741
Carretão II	Indigenous Land	1990	0.743
Karajá de Aruanã I	Indigenous Land	2000	0.104

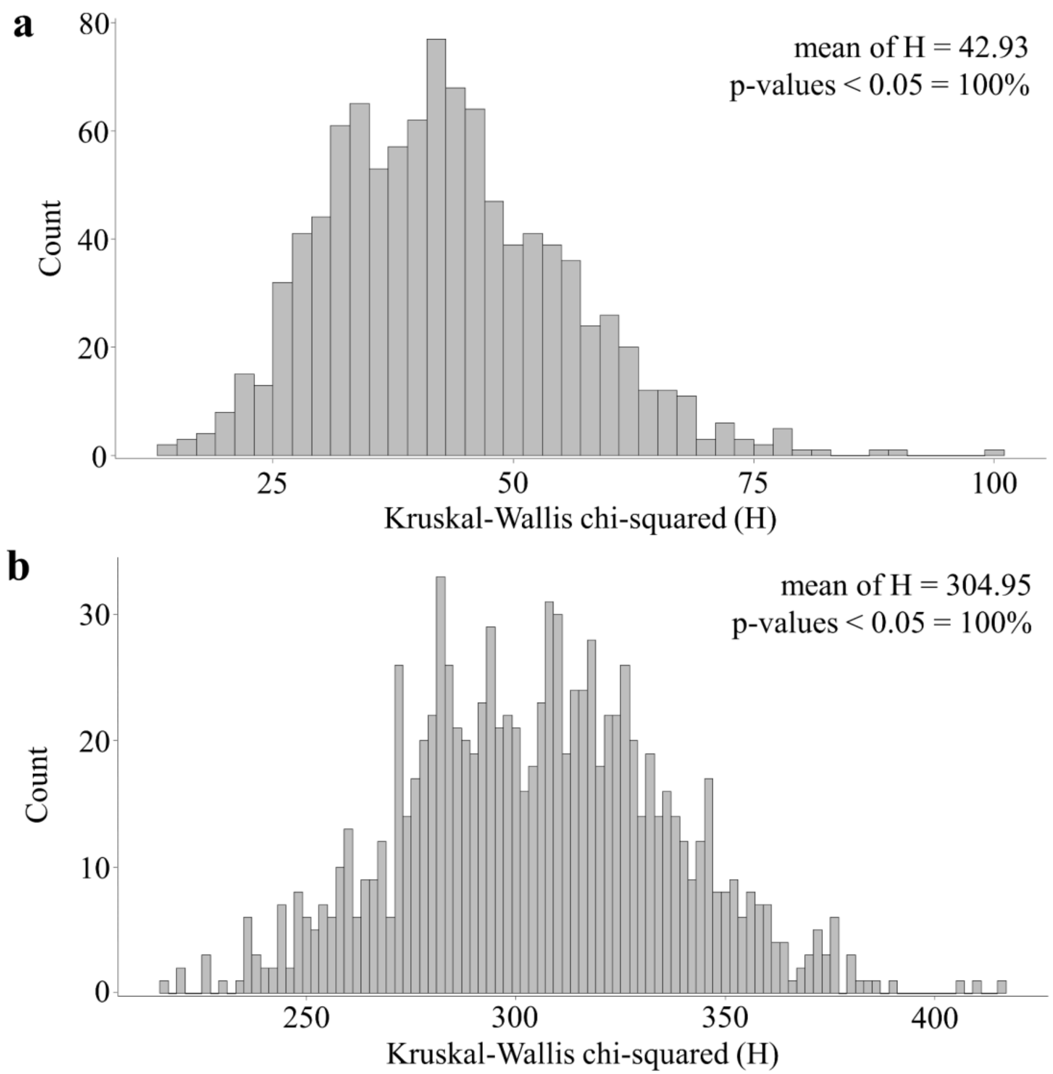
a
80
60
40
20
0
Count
mean of H = 42.93
p-values < 0.05 = 100%
25
50
75
100
Kruskal-Wallis chi-squared (H)
b
30
20
10
0
Count
mean of H = 304.95
p-values < 0.05 = 100%
250
300
350
400
Kruskal-Wallis chi-squared (H)

Figure S5. Histograms representing the Kruskal-Wallis H statistics dataset, generated by the resampling-based non-parametric approach, comparing the different protection levels in terms of environmental restoration (a) and degradation (b).
